# Population structure and dispersal routes of an invasive parasite, *Fascioloides magna,* in North America and Europe

**DOI:** 10.1186/s13071-016-1811-z

**Published:** 2016-10-13

**Authors:** Ludmila Juhásová, Ivica Králová-Hromadová, Eva Bazsalovicsová, Gabriel Minárik, Jan Štefka, Peter Mikulíček, Lenka Pálková, Margo Pybus

**Affiliations:** 1 Institute of Parasitology, Slovak Academy of Sciences, Hlinkova 3, 040 01 Košice, Slovakia; 2 Department of Molecular Biology, Faculty of Natural Sciences, Comenius University in Bratislava, Ilkovičova 6, Mlynská dolina, 842 15 Bratislava Slovakia; 3 Institute of Molecular Biomedicine, Faculty of Medicine, Comenius University, Sasinkova 4, 811 08 Bratislava, Slovakia; 4 Geneton Ltd, Ilkovičova 3, 841 04 Bratislava, Slovakia; 5 Biology Centre CAS, Institute of Parasitology and Faculty of Science, University of South Bohemia, Branišovská 31, 370 05 České Budějovice, Czech Republic; 6 Department of Zoology, Faculty of Natural Sciences, Comenius University in Bratislava, Ilkovičova 6, Mlynská dolina, 842 15 Bratislava Slovakia; 7 Alberta Fish and Wildlife Division and Department of Biological Sciences, University of Alberta, 6909-116 St, Edmonton, AB T6H 4P2 Canada

**Keywords:** Microsatellites, Parasite, Giant liver fluke, *Fascioloides magna*, Genetic interrelationships, Migratory routes

## Abstract

**Background:**

*Fascioloides magna* (Trematoda: Fasciolidae) is an important liver parasite of a wide range of free-living and domestic ruminants; it represents a remarkable species due to its large spatial distribution, invasive character, and potential to colonize new territories. The present study provides patterns of population genetic structure and admixture in *F. magna* across all enzootic regions in North America and natural foci in Europe, and infers migratory routes of the parasite on both continents.

**Methods:**

In total, 432 individuals from five North American enzootic regions and three European foci were analysed by 11 microsatellite loci. Genetic data were evaluated by several statistical approaches: (i) the population genetic structure of *F. magna* was inferred using program STRUCTURE; (ii) the genetic interrelationships between populations were analysed by PRINCIPAL COORDINATES ANALYSIS; and (iii) historical dispersal routes in North America and recent invasion routes in Europe were explored using MIGRATE.

**Results:**

The analysis of dispersal routes of the parasite in North America revealed west-east and south-north lineages that partially overlapped in the central part of the continent, where different host populations historically met. The exact origin of European populations of *F. magna* and their potential translocation routes were determined. Flukes from the first European focus, Italy, were related to *F. magna* from northern Pacific coast, while parasites from the Czech focus originated from south-eastern USA, particularly South Carolina. The Danube floodplain forests (third and still expanding focus) did not display relationship with any North American population; instead the Czech origin of the Danube population was indicated. A serial dilution of genetic diversity along the dispersion route across central and eastern Europe was observed. The results of microsatellite analyses were compared to previously acquired outputs from mitochondrial haplotype data and correlated with past human-directed translocations and natural migration of the final cervid hosts of *F. magna*.

**Conclusions:**

The present study revealed a complex picture of the population genetic structure and interrelationships of North American and European populations, global distribution and migratory routes of *F. magna* and an origin of European foci.

**Electronic supplementary material:**

The online version of this article (doi:10.1186/s13071-016-1811-z) contains supplementary material, which is available to authorized users.

## Background

Invasions and introductions of non-native (syn. non-indigenous, alien or exotic) species into novel localities are processes that have dramatically affected ecosystems worldwide. Human-caused biological introductions are a major force in shaping global biodiversity [1, 2] and often result in significant environmental changes and high economic costs [3], as well as reductions in biodiversity at local and regional scales [4].

The introduction of invasive parasites is usually an unintentional result of introduction of their final or intermediate hosts through international trade, global transportation, markets or tourism [5, 6]. Parasitic species become invasive if they are introduced to new territories, establish new populations, and spread rapidly into new habitats [7]. Negative effects caused by invasive parasites may be observed and studied based on epizootiological changes and current health status of affected host organisms. In addition, the population genetic characteristics of invasive parasites may change rapidly during the course of invasion into new habitats, following processes of founder effect or local adaptation [8].

A suitable model for above mentioned studies is the veterinary important parasite giant liver fluke, *Fascioloides magna* (Bassi, 1875) (Trematoda: Fasciolidae), which was transferred along with its cervid hosts from the Nearctic zone to the Palaearctic, where it established local populations [9]. Giant liver fluke represents a very remarkable species (with maximum length up to 8–10 cm), mainly due to its broad spatial distribution, invasive character, and potential to colonize new territories, which predestined it to become a centre of interest of veterinarians, parasitologists and population geneticists. The parasite utilizes a wide spectrum of free-living and domestic ruminants as final hosts, and aquatic snails as intermediate hosts. The pathological effects of *F. magna* infections depend mainly on the type of its final host. Red deer (*Cervus elaphus*), white-tailed deer (*Odocoileus virginianus*), caribou (*Rangifer tarandus*) and wapiti (*Cervus elaphus canadensis*) (definitive hosts) tolerate giant liver fluke very well and contribute significantly to maintaining its populations. Fascioloidosis in domestic ruminants and majority of free-living ruminants (aberrant and dead-end types of final hosts) may have a lethal effect [9].

It is generally accepted that *F. magna* is of North American (NA) origin where it co-evolved with an ancestral cervid host, *Odocoileus* spp. Currently, the parasite occurs in five major enzootic areas across the United States and Canada: (i) northern Pacific coast (NPC); (ii) Rocky Mountain trench (RMT); (iii) northern Quebec and Labrador (NQL); (iv) Great Lakes region (GLR); and (v) Gulf coast, lower Mississippi, and southern Atlantic seaboard (SAS) (for details see [9, 10]).

After its introduction to Europe, the giant liver fluke established three natural foci of infection: (i) north-western Italy (IT); (ii) Czech Republic and south-western Poland (CZ-PL); and (iii) Danube floodplain forests (DFF) (for details see [11, 12]). The first IT focus was established in the Regional Park La Mandria as a result of the documented import of wapiti from western USA in 1865 [13]. The southern and central parts of Czech Republic (first reference by Ullrich [14]) were recognized as the second focus of fascioloidosis, until recent confirmation of *F. magna* in the Lower Silesian Wilderness in the south-western Poland, close to the Czech border [15]. Since mitochondrial (mt) analysis confirmed that Czech and Polish samples represent the same genetic pool [12], this focus is recognized herein as CZ-PL. The third and still expanding focus occurs is the Danube floodplain forests (first documented in the 1990s), which represents a unique habitat located on islands of the inland delta of the Danube River, lacks ecological or human barriers for the movement of cervids and consequent dispersal of infective stages of their parasitic agents. In Europe, *F. magna* is considered to be an invasive species with high potential to spread and colonize new geographic territories and establish local populations. Therefore, the population genetics of *F. magna* has emerged as an interesting topic for investigation.

Non-recombining, maternally inherited mitochondrial DNA (mtDNA) was applied in our previous population studies on *F. magna* [10, 11] as a first, easy to obtain marker of choice. The study of the European *F. magna* populations revealed that: (i) IT population was specific and distinct from two other EU populations; (ii) flukes from IT clustered with parasites from RMT and NPC; western NA origin of the IT population was confirmed; an exact origin of the IT focus was not specified; (iii) flukes from DFF were closely related to CZ parasites and Czech origin of the Danube population was suggested; (iv) *F. magna* from CZ and DFF clustered with parasites from SAS; south-eastern US origin of the CZ population was implied; an exact origin of the CZ focus was not specified; (v) multiple introductions of *F. magna* from NA to EU were confirmed [11]. In the North America, the phylogenetic analysis of mt haplotypes revealed two major *F. magna* clades, western (NPC and RMT) and eastern (GLR, SAS and NQL). This genetic makeup correlated with data on historical distribution of white-tailed deer in the eastern and wapiti in the western part of NA [10].

Although the analyses of mt haplotypes provided useful pilot information about the interrelationships of giant liver fluke on both continents, they did not reveal detailed population history and migratory routes of the parasite within and between the continents, as well as an exact origin of the European populations of *F. magna*. Mitochondrial DNA as a single locus molecular marker with specific structure and mode of inheritance has its limitations and reliance only on mtDNA markers in population genetic studies has been criticized, e.g. [16]. Besides, phylogeographic analyses based solely on the mtDNA are known to reveal only a limited part of the evolutionary history of a species and application of multilocus nuclear markers, such as microsatellites (or short tandem repeats, STR), is often recommended [17]. Microsatellites are biparentally inherited nuclear markers that undergo recombination, thereby integrating additional genealogical processes [18]. Recently, we identified novel microsatellite loci candidates, designed primers and developed multiplex panels for their routine utilization in *F. magna* population studies [19].

The aim of the present study was to apply a multilocus approach to improve understanding of the patterns of population genetic structure and admixture in *F. magna* across all enzootic regions in North America and natural foci in Europe. We were interested in particular topics: (i) Would microsatellite data confirm the differentiation of North American *F. magna* populations into western and eastern lineages, as previously determined by mt haplotypes? (ii) Compared with mtDNA markers, microsatellites possess different genetic characteristics (e.g. co-dominance, polymorphism, multi-locus markers, Mendelian inheritance), hence, would they provide more detailed structuring of NA populations of the giant liver fluke? (iii) Since previous studies indicated that migration pattern of *F. magna* in NA correlates with the distribution and movements of cervids, we tested alternative hypotheses of historical population migration in NA allowing several competing colonization scenarios and compared the results with data on migration routes of definitive hosts; (iv) Would microsatellites confirm the population structure of EU *F. magna* populations as determined by mtDNA? (v) While mt haplotypes specified the origin of EU populations of *F. magna* only at the level of enzootic regions, do microsatellites promise a determination of the exact origin (particular US state/Canadian province) of European populations? (vi) Does the dispersal history of introduced EU populations correlate with chronological findings of the parasite according to the literature data? The results on the patterns of population structure derived from an extensive dataset of alleles for 11 microsatellite loci were discussed with outputs recovered previously from mt haplotypes. Also, the data on migration pattern of the giant liver fluke were compared with data on human-directed translocation and natural migration of cervid hosts of *F. magna*.

## Methods

### Parasite collection

Adult *F. magna* were isolated from fibrous pseudocysts in the liver parenchyma of final hosts, rinsed in PBS buffer and preserved in 96 % ethanol. We analysed 432 individuals from 38 final hosts from all North American (NA) enzootic regions (Table 1) and 74 cervids from all European (EU) natural foci (Table 2). Details on hosts, Canadian provinces, US states and European countries are provided in Tables 1 and 2.

**Table 1:** Details on final hosts and geographical localities of *Fascioloides magna* from North America analysed in the present study

N^a^	N^b^	Liver code	Final host	Locality	Geographical coordinates	USA state/ CA province	Enzootic region
3	3	OR-33	*Odocoileus hemionus columbianus* (black-tailed deer)	Salem	44°56′N, 123°02′W	USA Oregon (OR)	northern Pacific coast (NPC)
4	3	BC-1	*Cervus elaphus roosevelti* (Roosevelt elk)	Vancouver Island	49°00′N, 127°00′W	Canada British Columbia (BC)	
	1	BC-2					
89	10	AB-1	*Cervus elaphus* *canadensis* (wapiti)	Banff National Park	51°12′N, 115°35′W	Canada Alberta (AB)	Rocky Mountain trench (RMT)
	3	AB-2					
	6	AB-3					
	3	AB-4					
	4	AB-6					
	9	AB-7					
	8	AB-9					
	8	AB-11					
	11	AB-12					
	7	AB-13					
	3	AB-14					
	1	AB-15					
	3	AB-17					
	4	AB-18					
	1	AB-1a					
	2	AB-2a					
	2	AB-3a					
	2	AB-4a					
	2	AB-5a					
12	1	QC-1	*Ovibos moschatus* (muskox)	Kuujjuaq Tasiujaq	58°44′N, 70°02′W 58°06′N, 68°23′W	Canada Quebec (QC)	northern Quebec and Labrador (NQL)
	5	QC-2					
	4	QC-3					
	2	QC-4					
1	1	NL-3	*Rangifer tarandus* (caribou)	northern Labrador Nashaupi River	53°55′N, 60°44′W	Canada Labrador (NL)	
14	1	MN-1	*Odocoileus virginianus* (white-tailed deer)	Erskine, Hibbing	47°40′N, 96°00′W	USA Minnesota (MN)	Great Lakes region (GLR)
4	4	GA-31	*O. virginianus* (white-tailed deer)	Georgia	32°48′N, 83°09′W	USA Georgia (GA)	Gulf Coast, Lower Mississippi and southern Atlantic seaboard (SAS)
3	3	MS-35	*O. virginianus* (white-tailed deer)	River Valley	32°32′N, 91°22′W	USA Mississippi (MS)	
15	4 11	FL-36 FL-37	*O. virginianus* (white-tailed deer)	White Oak plantation	30°44′N, 81°45′W	USA Florida (FL)	
3	3	LA-39	*O. virginianus* (white-tailed deer)	Tensas National Wildlife Refuge	32°03′N, 91°15′W	USA Louisiana (LA)	
28	5	SC-40	*O. virginianus* (white-tailed deer)	Savannah River Site	34°26′N, 82°51′W	USA South Carolina (SC)	
	5	SC-41					
	8	SC-42					
	7	SC-43					
	3	SC-45					
176		38	total				

*N* ^*a*^ number of *F. magna* specimens from the respective USA state or Canadian (CA) province

*N* ^*b*^ number of flukes analysed from the respective final host individual

**Table 2:** Details on final hosts and geographical localities of *Fascioloides magna* from Europe analysed in the current study

N^a^	N^b^	Liver code	Final host	Locality	Geographical coordinates	European country	Natural focus
51	3	IT-1	*Cervus elaphus* *elaphus* (red deer)	Regional Park La Mandria Turin	45°7′N, 7°38′E	Italy (IT)	Italy (IT)
	3	IT-2					
	2	IT-3					
	6	IT-4					
	5	IT-5					
	3	IT-7					
	1	IT-8					
	3	IT-10					
	4	IT-11					
	5	IT-12					
	1	IT-13					
	1	IT-14					
	1	IT-15					
	2	IT-16					
	1	IT-17					
	2	IT-18					
	3	IT-19					
	4	IT-20					
	1	IT-21					
101	1	CZ-1	*C. e. elaphus* (red deer)	Křivoklát Mountains, Křivoklát	50°02′N, 13°52′E	Czech Republic (CZ)	Czech Republic and south-western Poland (CZ-PL)
	3	CZ-2		Brdy Mountains, Mirošov	49°41′N, 13°39′E		
	1	CZ-3		Brdy Mountains, Brdy	49°43′N, 13°55′E		
	8	CZ-4		Brdy Mountains, Beroun	49°57′N, 14°04′E		
	12	CZ-5		Křivoklát Mountains, Křivoklát	50°02′N, 13°52′E		
	1	CZ-6		Bohemian Forest, Chvalšiny	48°52′N, 14°11′E		
	4	CZ-7		Bohemian Forest, Chvalšiny	48°52′N, 14°11′E		
	2	CZ-8		Bohemian Forest, Květušín	48°47'N, 14°08'E		
	5	CZ-9		Brdy Mountains, Mirošov	49°41′N, 13°39′E		
	5	CZ-10		Bohemian Forest, Nové Údolí	48°49′N, 13°47′E		
	4	CZ-11		Brdy Mountains, Mirošov	49°41′N, 13°39′E		
	2	CZ-12		Bohemian Forest, Chvalšiny	48°52′N, 14°11′E		
	3	CZ-13		Brdy Mountains, Mirošov	49°41′N, 13°39′E		
	4	CZ-14		Hostýnske Mountains, Chvalčov	49°23′N, 17°42′E		
	2	CZ-15		Bohemian Forest, Květušín	48°47'N, 14°08'E		
	4	CZ-16		Bohemian Forest, Květušín	48°47'N, 14°08'E		
	2	CZ-17		Hostýnske Mountains, Chvalčov	49°23′N, 17°42′E		
	4	CZ-18		Bohemian Forest, Prášily	49°06′N, 13°23′E		
	2	CZ-19		Bohemian Forest, Chvalšiny	48°52′N, 14°11′E		
	4	CZ-20		Bohemian Forest, Horní Planá	48°46′N, 14°01′E		
	2	CZ-21		Bohemian Forest, Horní Planá	48°46′N, 14°01′E		
	3	CZ-22		Bohemian Forest, Horní Planá	48°46′N, 14°01′E		
	3	CZ-23		Bohemian Forest, Chvalšiny	48°52′N, 14°11′E		
	3	CZ-24		Bohemian Forest, Horní Planá	48°46′N, 14°01′E		
	4	CZ-25		Bohemian Forest, Chvalšiny	48°52′N, 14°11′E		
	5	CZ-26		Brdy Mountains, Mirošov	49°41′N, 13°39′E		
	4	CZ-27		Bohemian Forest, Boletice	48°49′N, 14°13′E		
	4	CZ-28		Bohemian Forest, Chvalšiny	48°52′N, 14°11′E		
4	1	PL-1	*C. e. elaphus* (red deer)	Lower Silesian Wilderness (south-western Poland)	51°22′N, 15°07′E	Poland (PL)	
	1	PL-2					
	1	PL-3					
	1	PL-5					
46	3	SR-1	*C. e. elaphus* (red deer)	Čičov	47°46′N, 17°46′E	Slovakia (SK)	Danube floodplain forests (DFF)
	14	SR-2		Kravany	47°46′N, 18°29′E		
	16	SR-16		Bodíky	47°55′N, 17°27′E		
	13	SR-22		Bodíky	47°55′N, 17°27′E		
20	2	HU-1	*C. e. elaphus* (red deer)	Szigetkӧz	47°51′N, 17°27′E	Hungary (HU)	
	2	HU-2					
	2	HU-3					
	2	HU-4					
	2	HU-5					
	2	HU-6					
	2	HU-7					
	2	HU-8					
	2	HU-9					
	2	HU-10					
34	1	CR-4	*C. e. elaphus* (red deer)	Slavonia, Tikveš	45°40′N, 18°51′E	Croatia (CR)	
	1	CR-6					
	2	CR-10					
	6	CR-12					
	7	CR-13					
	6	CR-14					
	7	CR-15					
	1	CR-17					
	3	CR-23					
256		74	total				

*N* ^*a*^ number of *F. magna* specimens from the respective European country

*N* ^*b*^ number of flukes analysed from the respective final host individual

### Isolation of genomic DNA

Genomic DNA was isolated from 20 mg of adult flukes using phenol:chlorophorm:isoamyl alcohol extraction and ethanol precipitation [20]. In order to completely remove remaining PCR inhibitors, such as divalent cations and proteins, two additional wash steps using the QIAamp® DNA Kit (QIAGEN, Hilden, Germany) were employed in the DNA purification procedure. Finally, genomic DNA was diluted in deionised water and stored at -20 °C.

### *Fascioloides magna*-specific primers for PCR amplification of 11 microsatellite loci

Primers for microsatellite loci were identified and validated by Minárik et al. [19] (see Additional file 1: Table S1). Prior to PCR amplification with fluorescently labelled primers and fragment analysis, standard PCR amplification for all 11 microsatellite assays was performed in order to eliminate individuals with degraded DNA displayed by a loss of specific primer binding sites or degradation of amplification targets.

In total, 20 μl of amplification mixture contained 10–20 ng of gDNA, 10 μl 2× PCR Master Mix (Fermentas, UAB, Vilnius, Lithuania) and 10 pmol of each of the primers (Additional file 1: Table S1). The conditions of PCR reactions were: 5 min at 94 °C, followed by 30 cycles of 1 min at 94 °C, 1 min at 55 °C, and 2 min at 72 °C. The final step was 5 min at 72 °C. The amplified PCR products were visualised on 1 % agarose gels.

### Fragment analysis in multiplex panels

Amplification conditions in multiplex PCR assays were the same as used for PCR amplification except that one of the primers in each primer pair was fluorescently labelled. The 432 individuals with expected amplification were analysed in four multiplex PCR panels and fragment analysis was performed as described in Minárik et al. [19]. The GENEMAPPER v.3.7 software (Applied Biosystems) was used for genotyping samples.

### Inference of population genetic structure and statistical evaluations

Statistical parameters, such as numbers of different (Na) and effective (Ne) alleles, their frequencies in populations, observed heterozygosity (Ho), expected heterozygosity (He) and deviation from Hardy-Weinberg equilibrium (HWE) were calculated using GENALEX 6.5 [21, 22]. Linkage Disequilibrium (LD) among pairs of loci within and between populations was determined using GENEPOP 4.2 [23]. The *P*-values obtained were adjusted by sequential Bonferroni correction. Deviations from HWE were measured by the coefficient of inbreeding, FIS [24]. The significance of LD between pairs of loci was computed using the Fisher’s exact test through 10^4^ iterations. The presence of null alleles was estimated for all eight populations of *F. magna* by MICRO-CHECKER 2.2.3 [25].

The programme BOTTLENECK [26] was applied to test whether introduced EU populations of *F. magna* (IT, CZ and DFF) underwent bottlenecks. PL population was not included in the analysis due to the low number of sampled specimens, thus CZ-PL focus was represented only by Czech samples. In addition, we assessed NA populations which were closely related to the European populations (RMT and SAS). RMT was used for a comparison with IT due to a low number of samples from the NPC population. Coalescent simulations were applied to generate gene diversities for each population and locus expected from the observed number of alleles given the sample sizes and assuming mutation-drift equilibrium. Expected and observed gene diversities were compared to assess whether gene diversity excess or deficit at each locus was present. The stepwise mutation model (SMM) and the two-phase model (TPM) of microsatellite evolution were employed in making calculations using Wilcoxon signed-rank tests [27] with 2,000 iterations. Under the TPM of mutation, 95 % single-step mutations were used [28].

The population genetic structure of *F. magna* was inferred based on a Bayesian clustering approach implemented in STRUCTURE 2.3.4 [29], a model-based method for estimation of the proportion of admixture from multilocus genotypes. Following the indications of the MICROCHECKER analysis, the possible occurrence of null alleles could not be excluded for some populations. Consequently, null alleles were allowed for all 11 loci. Posterior probabilities for the number of clusters K (log likelihood, Ln) as well as the *ad hoc* statistic ΔK [30] were estimated for values of K from 1 to 10 using an admixture model with uncorrelated allele frequencies. A series of 10 independent runs for each K, with 10^6^ MCMC repetitions, following a burn-in period of 100,000 iterations was conducted to test the accuracy of results [31]. For implementation of the ΔK statistic, the STRUCTURE HARVESTER [32] was applied.

In addition to Bayesian clustering, PRINCIPAL COORDINATES ANALYSIS (PCoA) was used to analyse the genetic interrelationships between global *F. magna* populations using GENALEX 6.5 [21, 22]. PCoA was carried out using a matrix of pair-wise genetic distances between individuals calculated [33]. A phylogenetic tree based on allele frequency data was constructed using the neighbour-joining method in POPTREE2 [34]. Bootstrap test was calculated with 1,000 replicates.

Historical dispersal routes in NA and recent invasion routes in EU were explored using a Bayes factor approach [35] in MIGRATE 3.6.10 [36, 37]. Ten alternative models of historical population migration in NA were tested. These models included five enzootic areas and parameter estimation was usually reduced to a single direction of gene flow with very little or no reticulation between areas (Additional file 2: Table S4 and Figure S2). In agreement with the generally held view (e.g. [38, 39]) these models allowed for several variants of postglacial expansion from south to north on both east and west coasts with an overlap in the central-north (GLR and RMT). Competing scenarios of west to east and east to west migration were also tested, as well as a full model allowing migration between all population pairs.

Since MIGRATE was successfully applied to identify the source of a freshwater tapeworm recently introduced to northern Africa [40], we used it here to assess the invasion route of CZ and DFF populations of *F. magna*. Due to low number of samples, the PL population was not included in the analysis. We simulated several models of gene flow: (i) following the chronology of literature records on the spread of flukes, we modelled an invasion from the Czech Republic to Slovakia and then from Slovakia along the Danube to Hungary and Croatia; (ii) alternatively, we modelled Slovakia as the source and the Czech Republic, Hungary and Croatia as the recipients; and (iii) we reversed the direction of the invasion starting from Croatia towards Hungary, Slovakia and Czech Republic. The full model allowing migration between all population pairs also was tested. All models were compared and ranked using Bayes Factor analysis based on marginal likelihoods obtained in the MIGRATE analysis. MIGRATE was run using Bayesian inference and Brownian motion approximations of the stepwise microsatellite mutation model. Each run comprised 5 parallel chains with temperatures 1.0, 1.5, 3.0, 100 and 1,000,000 and each run was replicated 10 times. Run parameters were: 300,000 MCMC steps burn-in, sampling 3,000 parameters every 1,000 steps for the NA dataset and 200,000 MCMC steps burn-in, sampling 2,000 parameters every 1,000 steps for the European dataset. Prior distributions were uniform with a range of 0.00 to 60.00 for Theta (*Θ*) and 0.00 to 1,000.00 for migration rate (*M*).

Complementary to the BOTTLENECK analysis, in MIGRATE we also estimated relative population sizes (*Θ*) of EU and NA populations identified as their potential source (NPC *versus* IT; South Carolina *versus* CZ, SK, HU and CR) to quantify the impact of bottlenecks. PL was not included due to the small sample size. We also assessed the possibility of capturing recent bottleneck events using the skyline plot option for *Θ*. MIGRATE was run using the same settings as above, using the winning model of migration direction for the EU dataset.

## Results

### Basic statistics of STR loci

All examined microsatellite loci had variable mean numbers of different alleles (Na) per locus, ranging from 2.636 (NPC) to 6.818 (SAS) (Table 3). The highest effective allelic richness (Ne) was detected in SAS (3.734), the least mean number of effective alleles was in IT (1.845). While in RMT, SAS, CZ-PL and DFF multiple cases of potential null alleles were detected, much fewer loci potentially affected by null alleles were identified in NPC, NQL, GLR and IT populations, where the estimated frequency of null alleles was generally low (Additional file 3: Tables S2 and Additional file 4: Table S3). The monomorphic loci were observed in NPC and NQL (NA; Additional file 3: Table S2), and in IT (EU; Additional file 4: Table S3). The presence of monomorphic loci in IT is probably due to restricted gene flow within this geographically closed region.

**Table 3:** Descriptive statistics for 11 microsatellite loci of *Fascioloides magna* from North America and Europe

Region/focus^a^		Na	Ne	Ho	uHe	F	%P
NPC	mean	2.636	1.939	0.260	0.375	0.283	
	SE	0.527	0.384	0.090	0.088	0.146	
							73
RMT	mean	6.182	2.822	0.446	0.584	0.203	
	SE	1.077	0.340	0.052	0.064	0.048	
							100
NQL	mean	3.000	1.944	0.357	0.353	−0.070	
	SE	0.647	0.348	0.097	0.093	0.072	
							73
GLR	mean	5.455	3.682	0.588	0.656	0.124	
	SE	0.835	0.599	0.088	0.069	0.117	
							100
SAS	mean	6.818	3.734	0.474	0.648	0.282	
	SE	1.271	0.654	0.070	0.055	0.083	
							100
IT	mean	3.091	1.845	0.335	0.337	0.020	
	SE	0.667	0.294	0.087	0.083	0.065	
							82
CZ-PL	mean	5.727	3.524	0.581	0.655	0.142	
	SE	0.776	0.456	0.080	0.059	0.086	
							100
DFF	mean	3.636	2.070	0.401	0.459	0.190	
	SE	0.432	0.237	0.064	0.058	0.094	
							100

^a^Codes for North American enzootic regions and European natural foci are explained in Tables 1 and 2, respectively

*Abbreviations*: *Na* number of different alleles, *Ne* number of effective alleles, *Ho* observed heterozygosity, *uHe* unbiased expected heterozygosity, *F* fixation index, *%P* percentage of polymorphic loci, *SE* standard error

HW disequilibrium was found for multiple loci in RMT, SAS and CZ-PL populations (Additional file 3: Table S2 and Additional file 4: Table S3). Exact tests for LD (after sequential Bonferroni correction) showed significant *P*-values only in three populations: 12 pairs of loci in CZ population, 14 pairs in RMT and 43 pairs in SAS populations (results not shown). HW and LD patterns of disequilibria in RMT, SAS and CZ-PL pointed to possible population substructuring or recent admixture, while in the other NA regions and EU foci inner population substructuring is probably absent. Potential sutures in population structure, independent of *a priori* population determination, were further explored using Bayesian assignment in STRUCTURE and clustering analysis in PCoA.

### Population genetic structure of *F. magna* in North America

**Fig. 1:**
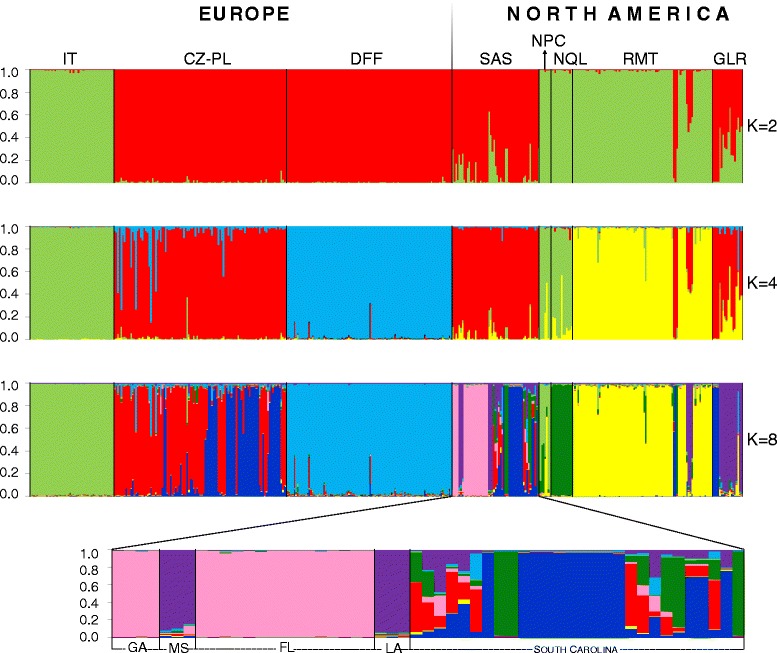
Bar plots from program STRUCTURE assigning all 432 *Fascioloides magna* individuals from North America (NA) and Europe (EU) into K = 2, 4 and 8 clusters and detailed structure of SAS region. Codes for NA enzootic regions, US states/Canadian province and EU natural foci are explained in Tables 1 and 2, respectively

Several statistical approaches were used for detection of genetic relationships among NA populations of *F. magna*. STRUCTURE analysis revealed two distinct genetic clusters (K = 2) according to the ΔK statistics (Additional file 5: Figure S1); one cluster was composed of SAS and GLR enzootic regions, and a second cluster involved NPC, NQL and RMT populations (Fig. 1). Two smaller but discernible peaks in the ΔK statistics (K = 4 and K = 8) reflect further substructuring. With the increasing K values, the second cluster appeared to be more differentiated; while populations from NPC and NQL showed similar genetic structure, those from RMT were more diverse (Fig. 1; K = 4). All populations pointed to a high genetic divergence at K = 8 (Fig. 1) with each population possessing clusters specific for the respective region.

**Fig. 2:**
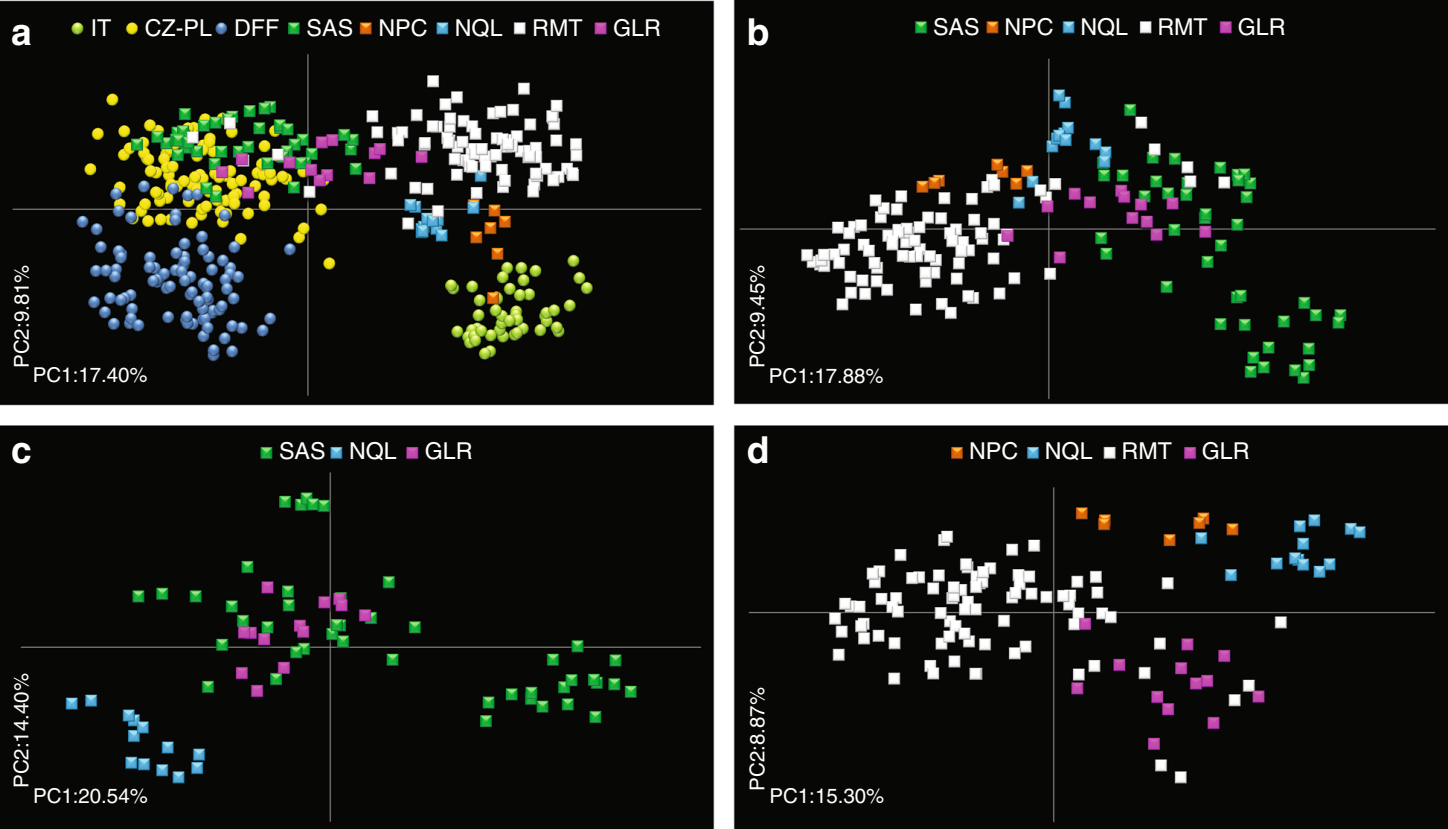
Genetic population structure of *Fascioloides magna* from all North American (NA) and European (EU) populations (**a**), and interrelationships among NA populations (**b**-**d**) derived from PRINCIPAL COORDINATES ANALYSIS (PCoA). Circles, EU natural foci; squares, NA enzootic regions. **a** All NA and EU populations. **b** All NA populations. **c** Eastern NA clade inferred from mt data (Bazsalovicsová et al. [10]). **d** West-east NA lineage derived from MIGRATE analysis of microsatellites (present study). Codes for NA enzootic regions and EU natural foci are explained in Tables 1 and 2, respectively

The PCoA analysis of 176 *F. magna* from NA revealed that flukes from some enzootic regions are geographically specific, while several populations interact with each other (Fig. 2). Genetically the most distinct groups of parasites were those from RMT and SAS with some individuals overlapping with NPC, NQL and GLR samples (Fig. 2b). In eastern populations (NQL, GLR and SAS) individuals from SAS and GLR clustered in adjoining assemblages with overlapping samples, while *F. magna* from NQL formed a separate distant set (Fig. 2c). Flukes from enzootic regions involved in the west-east NA lineage based on the MIGRATE analysis (see below) displayed admixture of flukes from RMT and GLR, while those from NQL and NPC formed marginal groups (Fig. 2d).

### Population genetic structure of *F. magna* in Europe

The genetic structure of the EU populations of *F. magna* was more straightforward and showed a simpler genetic pattern than NA populations. According to STRUCTURE, flukes from IT represented a distinct population at each K value with no genetic interconnection with any other European foci (Fig. 1). At K = 2, two distinct genetic clusters were evident; the first cluster comprised samples from the earliest known EU focus - IT, and the second cluster involved individuals from CZ-PL and DFF, which differed from each other at K = 4 (Fig. 1).

The Bayesian clustering approach under the STRUCTURE showed some extent of genetic similarity between CZ-PL and DFF (Fig. 1). The very unique pattern of the DFF cluster, as seen in the STRUCTURE outputs, is probably the consequence of genetic drift caused by a founder effect of individuals dispersed from the CZ-PL focus.

**Fig. 3:**
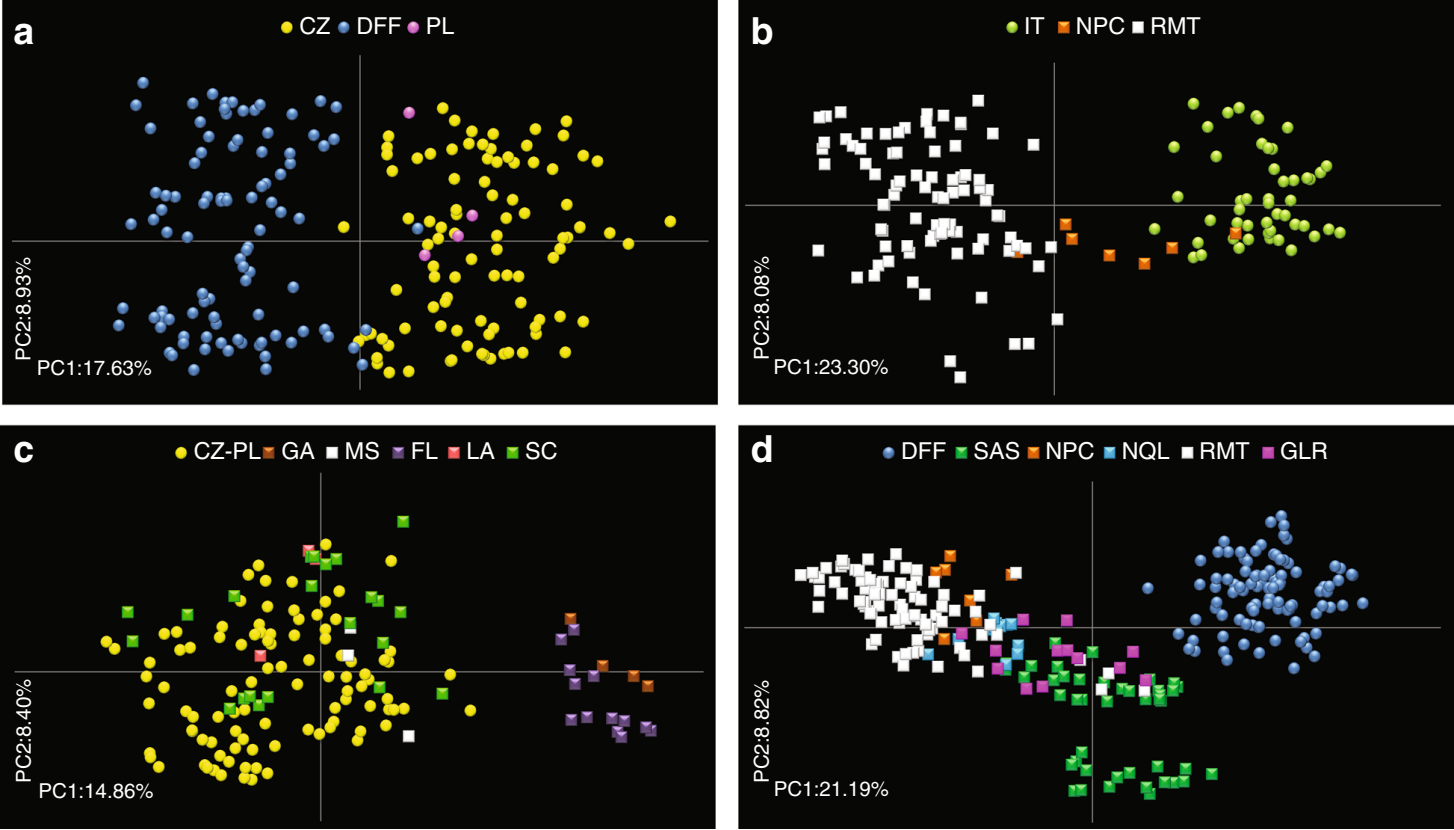
Genetic population structure of *Fascioloides magna* from European (EU) populations (**a**) and interrelationships between EU and North American (NA) populations (**b**-**d**) based on the PRINCIPAL COORDINATES ANALYSIS (PCoA). Circles, EU natural foci; squares, NA enzootic regions. **a** Interrelationships between *F. magna* from Czech-Polish (CZ-PL) and Danube floodplain forests (DFF) foci. **b** Relatedness between flukes from Italy (IT) and western NA regions (RMT and NPC). **c** Comparison between *F. magna* from CZ-PL and particular states of SAS. **d** Differentiation of DFF from all NA enzootic regions. Codes for NA enzootic regions and EU natural foci are explained in Tables 1 and 2, respectively

In accordance with STRUCTURE results, the PCoA analysis of 256 *F. magna* from Europe revealed that the IT population was most distinct from CZ-PL and DFF foci (Fig. 2a). The majority of individuals of CZ-PL and DFF formed distinct, non-overlapping assemblages with only very slight overlaps between them (Fig. 3a).

### Genetic interrelationships between *F. magna* from North America and Europe

Using STRUCTURE, the comparison of *F. magna* populations from both continents suggested close genetic interrelationships between IT samples and flukes from western (NPC and RMT) and eastern (NQL) NA regions (Fig. 1; K = 2). The interrelationship between IT and NA populations was more particular and specific with increasing the K-value. While at K = 4 the IT population was genetically similar to individuals from NPC and NQL, at K = 8 its close link to NPC emerged (Fig. 1). Comparison of flukes from IT and western NA regions (NPC and RMT) using PCoA analysis revealed particularly close genetic relationships between IT and NPC, while individuals from RMT did not show any genetic relatedness with Italian samples (Fig. 3b).

In present STRUCTURE and PCoA analyses, the SAS region was divided into individual US states (South Carolina, Florida, Georgia, Louisiana and Mississippi) for more detailed determination of genetic relationships. In STRUCTURE, the second distinct genetic cluster was formed by EU populations CZ-PL and DFF and eastern NA populations SAS and GLR (Fig. 1; K = 2). Flukes from CZ-PL displayed the highest genetic similarity with individuals from SAS; in particular those from South Carolina using both STRUCTURE (see the inset of SAS in Fig. 1) and PCoA (Fig. 3c) analyses. Hence, the exact origin of a second European focus was specified for the first time.

Comparing the DFF European focus with all NA populations in STRUCTURE analysis, flukes from DFF showed genetic distinctness already at K > 2 (Fig. 1). Similarly in PCoA analysis, *F. magna* from DFF did not cluster with any NA population (Fig. 3d).

**Fig. 4:**
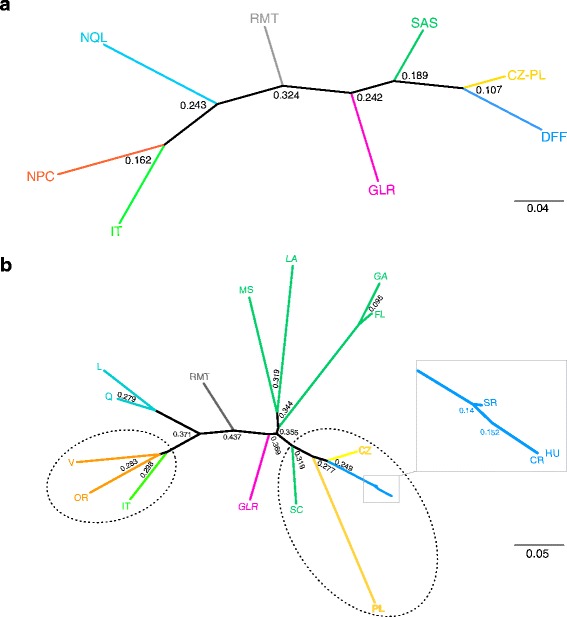
Unrooted neighbour-joining (NJ) evolutionary tree based on pairwise D_A_ distances among *Fascioloides magna* from Europe (EU) and North America (NA). **a** Tree representing *F. magna* from NA enzootic regions and EU natural foci. **b** Tree displaying *F. magna* from US states, Canadian provinces and EU countries coloured according to their respective region/locus. Dotted ovals, closest interrelationships between EU and NA populations. Codes for NA enzootic regions and EU natural foci are explained in Tables 1 and 2, respectively

**Fig. 5:**
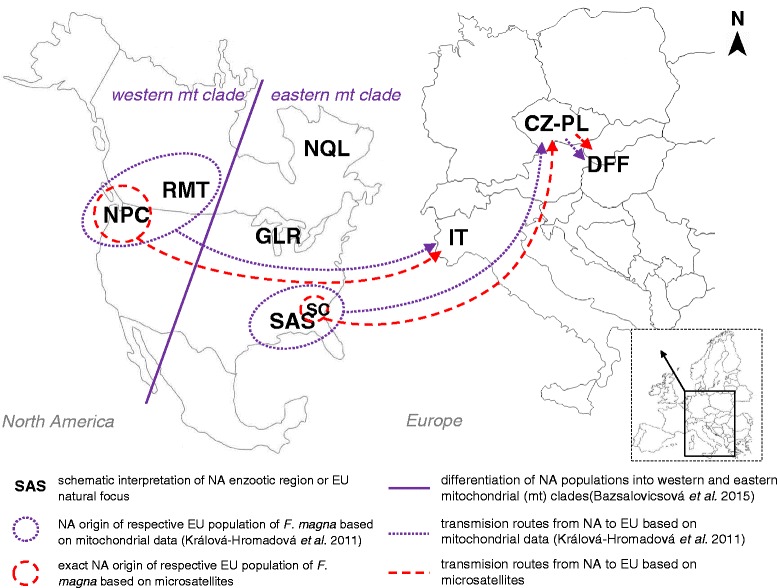
Schematic reconstruction of transmission routes between North American (NA) and European (EU) populations of *Fascioloides magna*, and determination of the origin of EU populations derived from mtDNA haplotypes (Králová-Hromadová et al. [11]) and microsatellite analysis

The phylogenetic analysis using POPTREE2 supported the outcomes of STRUCTURE and PCoA analyses. In the phylogenetic tree, IT formed a clade with samples from British Columbia and Oregon (NPC region), which confirmed western NA origin of *F. magna* from Italy and specified flukes from the NPC region to be genetically closest to the Italian ones (Fig. 4a). On the other hand, the representatives of CZ-PL and DFF showed closest relationships to those of SAS (Fig. 4a). Within SAS, flukes from SC displayed the closest relatedness to CZ-PL (Fig. 4b). Individual NA branches did not fully corroborate the mitochondrial determination of western (NPC and RMT) and eastern (SAS, GLR and NQL) separation. In the topology of the tree, the NQL branch occurred between NPC and RMT, distantly from GLR and SAS (Fig. 4a and b). Bootstrap nodal supports are generally low, but this is common for interpopulation analyses of microsatellite data. Figure 5 shows a schematic interpretation of transfer routes from NA to EU based on mt haplotypes [11], with more exact determination of origin revealed by current microsatellite data.

### Analysis of dispersal routes

**Fig. 6:**
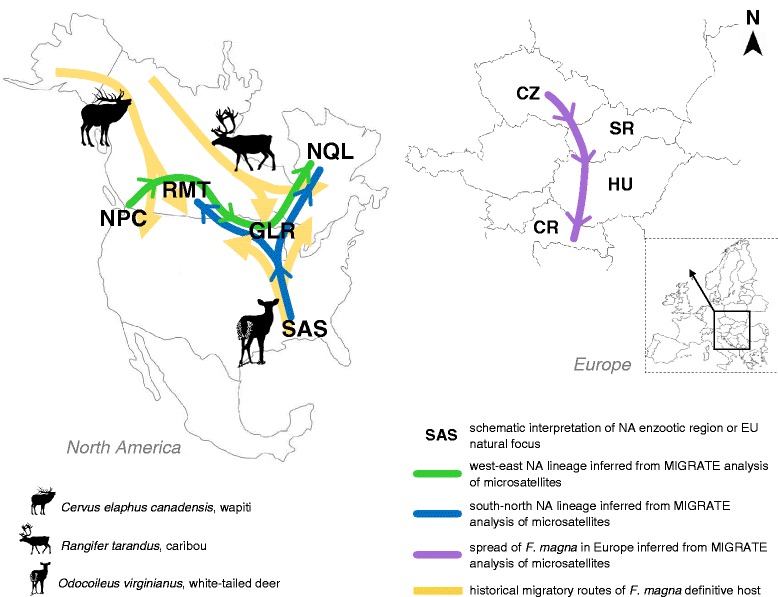
Schematic presentation of the most possible scenarios of migration routes of *F. magna* on both continents using MIGRATE

The testing of different hypotheses of dispersal routes of *F. magna* in NA using MIGRATE revealed the most probable scenario (Additional file 2: Table S4 and Table S5): (i) populations belonging to the west-east NA lineage dispersed along with its hosts in the direction NPC → RMT → GLR → NQL; and (ii) the south-north NA lineage suggested a second parallel migration in the direction SAS → GLR → NQL and SAS → GLR → RMT. The RMT and GLR regions appear to be regions of intersections of final host’s dispersal routes with consequent admixture of the fluke’s gene pool. In Europe, apart from IT with its restricted distribution, the expected hypothesis of dispersal route of *F. magna* was confirmed in the direction CZ → SK → HU → CR (Additional file 2: Table S4 and Table S5). Figure 6 provides a schematic interpretation of dispersal routes within NA and EU derived from MIGRATE analysis of microsatellite data.

### Bottleneck effect analysis

We found no statistical evidence that any of the five analysed populations (IT, CZ, DFF, SAS and RMT) were subjected to a recent bottleneck under the SMM and TPM mutation models using the BOTTLENECK software (Table 4). All populations exhibited some loci with higher than expected heterozygosity given the observed number of alleles. European populations revealed a higher proportion of loci with a heterozygosity excess (IT 44.4, CZ 63.6, DFF 54.5 and 45.5 %, respectively, depending on the mutation model) compared to NA populations (RMT and SAS; both 36.4 %). No population showed a significant (*P* < 0.05) deviation from equilibrium expectations (Table 4).

**Table 4:** Analyses of population size changes. Wilcoxon sign-rank tests for heterozygosity excess in native and introduced areas of *Fascioloides magna* under a two-phase model (TPM) and stepwise mutation model (SMM) from BOTTLENECK and coalescent estimates of Theta (*Θ*) for source and introduced *F. magna* populations

Focus/region^a^				
BOTTLENECK	TPM		SMM	
	Hex/Ht	*P*	Hex/Ht	*P*
RMT	4/11 (36.4 %)	0.320	4/11 (36.4 %)	0.102
IT	4/9* (44.4 %)	0.652	4/9* (44.4 %)	0.496
SAS	4/11 (36.4 %)	0.764	4/11 (36.4 %)	0.148
CZ	7/11 (63.6 %)	0.102	7/11 (63.6 %)	0.577
DFF	6/11 (54.5 %)	0.966	5/11 (45.5 %)	0.700
Focus/region				
MIGRATE	2.5 %	*Θ* mode	97.5 %	*Θ* mean
NPC	0	*1.620*	26.880	*10.249*
IT	0	*1.020*	2.120	*1.012*
SC	0.133	*3.667*	6.933	*3.615*
CZ	0	*2.200*	5.600	*2.278*
SK	0	*0.067*	3.600	*0.705*
HU	0	*0.067*	4.133	*0.988*
CR	0	*0.067*	4.667	*1.359*

^a^Codes for North American enzootic regions and European natural foci are explained in Tables 1 and 2, respectively

BOTTLENECK notes: *two loci out of 11 analysed were monomorphic in the IT population, *Hex/Ht* ratio of the number of loci with a heterozygosity excess to the total number of analysed polymorphic loci, *P* statistical significance of any deviation from equilibrium expectations

MIGRATE notes: 2.5 and 97.5 % demarcate the 95 % confidence interval of Theta estimates, *italicized numbers* the mode and mean values of Theta. MIGRATE estimates for NPC and IT, and for SC, CZ, SK, HU and CR originated from two separate runs

Comparison of the mean number of effective (Ne) and different (Na) alleles per population between native and invasive populations suggests a similar pattern (Table 3). In comparison between IT and NPC populations, the decrease of genetic diversity in the IT population was not substantial. The mean Ne value was detected to be only slightly lower for IT specimens (1.845) than seen in the parasites from NPC region (1.939). Concerning mean Na value, individuals from IT possessed even higher number (3.091) than flukes from NPC (2.636), although this is probably affected by larger sample size for IT. Similarly to the IT/NPC comparison, the bottleneck effect was also not evident between CZ-PL focus and SAS region in the BOTTLENECK analysis. The mean Ne and Na values were detected to be only slightly lower for the CZ-PL population (3.524/5.727) than seen in the parasites from SAS (3.734/6.818) (Table 3). Overall, lower Na values were detected in DFF compared with CZ-PL. The bottleneck effect was evident for the first time when considering the summary statistics; the mean Ne and Na values in DFF (2.070/3.636) were lower than in CZ-PL (3.524/5.727) (Table 3). Similarly, the values of observed heterozygosity (Ho) were lower in DFF compared to CZ-PL and the coalescent analysis showed smaller mean values of *Θ* compared to CZ.

On the contrary, comparison of relative population sizes using coalescent approach in MIGRATE revealed potential bottlenecks in all introduced populations. The mean estimate of *Θ* for IT was approximately one tenth of that for NPC (Table 4). Due to the genealogical approach based on coalescence of alleles MIGRATE estimates are less prone to statistical errors than frequentist methods when small sample sizes are used. Thus, we were able to compare the IT population directly with its suspected source (NPC), for which only seven specimens were available. According to microsatellite data, IT originated from a potentially already bottlenecked NPC population, as seen in the plots in the Additional file 2: Figure S2 and in a high proportion of monomorphic loci in NPC (Additional file 4: Table S3). However, coalescent approach in MIGRATE revealed a series of potential bottlenecks, first during the initial introduction to CZ and then during dispersal to the Danube floodplains area. It was shown that BOTTLENECK analysis approach may fail to uncover even known population contractions (e.g. [41]) and thus may have failed also here.

The estimates for SC and the central and eastern EU populations showed a sequential drop of *Θ* values (Table 4). It should be noted that despite the differences in the mean values, majority of populations overlapped in the lower end of the 95 % confidence intervals of the estimates of *Θ*, only the SC population showed a value significantly higher than zero. Unfortunately, the relatively recent introduction events were not captured with sufficient precision by the skyline plot visualisation (Additional file 2: Figure S2). Despite the plots of population size changes through time that showed very recent coalescence in introduced populations compared to the source populations, and a gradual population decline seen in native NA populations, the confidence intervals surrounding mean values were too wide and did not allow unambiguous interpretation.

## Discussion

Using the giant liver fluke as a model, the present study was focused on revealing historical factors defining population genetic structure and admixture in parasites associated with large vertebrates, particularly those strongly affected by human activities. The current results identified different origins of *F. magna* populations in Europe, and a possible scheme of migratory routes of the parasite on its native North American continent. The microsatellite analyses revealed more detailed population structure than data acquired according to mt haplotypes [10, 11], and provided novel insights into the genetic interrelationships among *F. magna* populations, which were not apparent from mtDNA alone.

### Genetic interrelationships of North American populations of *F. magna*

Microsatellite data revealed the interrelationships between NA populations and their partial admixture, some of which differed from outcomes based on mtDNA. The first difference between mtDNA and microsatellite results regarded the western clade (NPC and RMT) [10], for which the microsatellites revealed a higher degree of heterogeneity. The more subtle population structuring can be explained by geographical and topographical separation of primary cervid hosts (wapiti) in the respective regions: NPC (represented by flukes from Oregon and British Columbia) is isolated from RMT (flukes from Banff National Park in western Alberta). These localities are separated by three mountain ranges (Coastal, Columbia and Rocky Mountains) that limit the interconnection of cervids between these regions, minimizes recent genetic admixture of flukes and leads to their region-specific genetic structure. In addition, the Banff population of the parasite is a result of relatively recent dispersal of wapiti across the Rocky Mountains and its subsequent rapid expansion on their western slopes [42], which may have facilitated distinct genetic separation of flukes from the shared gene pool with NPC.

Differences were also apparent within the eastern NA clade of *F. magna* (SAS, GLR and NQL) [10], where microsatellites confirmed close relatedness only between flukes from SAS and GLR. The northernmost and easternmost NQL population showed distinct genetic structure in all statistical approaches and displayed surprisingly close relationships with *F. magna* from the westernmost NPC region. This finding strongly opposes the results based on mtDNA, where the western and eastern populations were clearly separated with no overlaps. It is difficult to explain the genetic relationship of flukes coming from geographically isolated and remote localities at opposite sides of the continent, with no commercial or conservation reasons to translocate their cervid hosts. Hypothetically, these two populations may be the only remnants of the original ancient lineage of *F. magna* unadulterated by translocation or recent natural overlap. There could have been an ancient overlap with subsequent extirpation of host populations on the shared range and complete isolation of flukes in NQL in the far north-east and NPC in the far west. This could give rise to shared genetic makeup of *F. magna* in west (Oregon and British Columbia) and north-east (Quebec and Labrador), resulting in isolated remnant fluke populations with a shared ancestry.

Results of the MIGRATE analysis indicated two most probable paths of dispersal in NA, the west-east and the south-north NA lineages, which correlate with historical movement and current distribution of definitive hosts of *F. magna*, such as white-tailed deer, wapiti and caribou. While *F. magna* co-evolved with ancestral white-tailed deer *Odocoileus* spp. and was widespread in major wetland habitats throughout NA, wapiti or caribou sympatric with white-tails encountered giant liver fluke in overlapping contaminated wetlands [9]. Although white-tails were abundant in the south-eastern USA during the Pleistocene epoch [43], unrestricted hunting, deforestation and extensive agricultural development in that region in the 19th century let to their dramatic declines [44]. Implementation of an extensive restocking program during the late 19th and in 20th centuries increased deer populations especially in the south-east [45]. Currently, the white-tailed deer represents the most common and sought-after NA game animal with wide distribution from south-eastern USA toward southern Canada, especially east of Rocky Mountains [46]. Thus, the south-north direction of *F. magna* migration correlates with the distribution of white-tails in south-eastern USA and their northern migratory routes.

The second west-east NA lineage might be related to historical distribution of wapiti, which were established across NA south of the forested boreal zone from the Atlantic to the Pacific oceans. Wapiti occurred from northern British Columbia east to New York, and south to South Carolina [47]. They were extirpated from large parts of their historical range in late 19th and early 20th centuries, but later on they were reintroduced in parts of their native range and also to some areas outside of their known historical range [47]. A relatively small number of wapiti infected with *F. magna* were translocated from NPC in the early 1900s to Wainwright in Alberta (RMT); three decades later wapiti were relocated from Wainwright to GLR region [48]. This may have infused genetic makeup of *F. magna* from west into the eastern fluke population around the Great Lakes.

The admixture of parasite’s gene pool in the central GLR region, reflected also by positive HW and LD tests, is very probably related also to historical spatial distribution of white-tailed deer and caribou. Previous more northern geographic range of white-tailed deer and more southern of caribou resulted in shared pastures of both cervids in overlapping regions around Great Lakes. In the 19th century, peripheral populations of caribou within the USA were eradicated and populations that occurred from Minnesota to Maine, in New York, Wisconsin and Michigan were presumed to be extinct or disappeared [49]. The current fluke population in caribou in NQL is a residual population that survived the caribou extirpations in more southern regions, but managed to acquire *F. magna* in the overlapping GLR region and introduced it into the northernmost NQL. The GLR was apparently the region of intersections of final host’s dispersal routes where *F. magna* could have retained some ancestral genetic structure. At the same time, the GLR population probably has intermingled with flukes after translocation of infected white-tailed deer within eastern NA, and limited introduction of infected wapiti from the west.

### Genetic interrelationships and an origin of European populations of *F. magna*

Populations introduced from native areas and isolated in novel territories are in general characterized by reduced allelic richness, lower genetic diversity, significantly stronger influence of random genetic drift and bottleneck effect [50]. In *F. magna*, the structure of introduced populations provides a somewhat different picture.

The discrepancy between results obtained from different methods (allelic richness data, BOTTLENECK analysis, MIGRATE) could suggest that bottlenecks associated with founder effect were not severe enough to drastically reduce genetic variability in the introduced populations. Alternatively, the failure of BOTTLENECK to uncover population contractions could be affected by the occurrence of null alleles in some populations (RMT, SAS, CZ-PL and DFF; Additional file 3: Table S2 and Additional file 4: Table S3), because BOTTLENECK uses the ratio of the number of loci with a heterozygosity excess to the total number of analysed polymorphic loci for inference [26]. On the contrary, the coalescent based MIGRATE analysis should be less prone to such artefacts.

Retaining sufficient genetic variability is a crucial factor in successful establishment of the invasive species, which may then experience quick evolutionary changes associated with adaptations to the new habitat (e.g. [51]). In parasites with complex life cycles involving free-living and parasitic stages, such processes may happen by inclusion of novel intermediate or final hosts, which is also the case of *F. magna*. In Europe, the fluke has adapted to a broad spectrum of new species of aquatic snail as well as free-living ruminants (for a review see [52]). Recent studies showed that the volume of neutral and adaptive genetic diversity in invasive species may significantly differ [53]. Whilst we analysed only neutral variability, future studies should focus on genes interacting directly with the environment. For instance, variability in the genes allowing penetration into the snail hosts and defence against host immune system known from *Schistosoma*-snail interactions (e.g. [54]) are candidates for quick adaptive changes.

In BOTTLENECK analysis, the comparison between EU and NA populations revealed that the bottleneck effect was neither evident between IT/NPC nor between the CZ-PL focus and SAS region. Smaller, but statistically significant difference in population size was found between SC and CZ-PL populations, with probable continuation of the dilution of population diversity towards the Danube area. The pattern of serially pronounced bottlenecks on the trajectory from NA to CZ-PL and DFF corroborates the proposed scenario of fluke invasion in the central European area. The CZ population was established by direct import of (potentially) multiple infected host individuals and created a genetically relatively rich fluke population with high potential for further spread. Another important fact is that a high genetic diversity was observed both in mt haplotypes of *F. magna* infrapopulations (a set of parasite species coming from single host) [10] and in allelic variation of microsatellites (present study, data not shown). In some cases, the genetic polymorphism of infrapopulations of *F. magna* (mainly in SAS) even represented an overall level of variation detected in the respective region. Thus, cervids imported from NA harboured highly polymorphic *F. magna* infrapopulations, which established a genetically rich fluke population in the CZ focus.

Evidently a different situation happened during the introduction of *F. magna* from CZ-PL to DFF European foci. Outputs of population statistics (Table 3) and Bayesian inferences (MIGRATE, Table 4) congruently indicate reduced allelic richness and lower genetic diversity in the DFF population, as a result of genetic drift or bottleneck effect characteristic for a translocated population that underwent severe size reduction. Apparently only a few infected cervids harbouring a population of flukes with limited genetic variation were translocated from the CZ-PL focus, which resulted in a diluted gene pool in the DFF focus. There is no convincing evidence of a transmission mode of *F. magna* from Czech to Danube focus. Independently from the way of transmission, there was significant dilution of the genetic pool in DFF compared with the initial genetic stock in the Czech focus. In the case of natural dispersion of the parasite along with its hosts across the landscape, higher genetic diversity would be expected in the newly established population, contrary to human-mediated translocation of the parasite, which would result in reduced allelic richness and evidence of a bottleneck effect. Hence, current results on genetic structure imply man-made transport of *F. magna* from CZ-PL to DFF focus.

## Conclusions

In conclusion, current results achieved the initial expectations of our study. Microsatellites, as multi-locus markers characterized by Mendelian inheritance and higher level polymorphisms, provided more detailed population structuring and novel insights on North American and European populations of *F. magna* both within and between the continents. Although the microsatellite data mostly correlated with outputs recovered previously from mt haplotypes, some differences were revealed. In North America, a higher degree of heterogeneity was revealed for both western and eastern lineages, which is probably related to migration history of definitive cervid hosts. Similarly in Europe, the genetic structure of *F. magna* populations agreed with results previously determined by mtDNA; the Italian population was genetically distinct from two other European foci, CZ-PL and DFF. The novelty of the current study is determination of the exact origin of CZ-PL and IT natural foci for the first time. The most significant and original benefit of microsatellite data is undoubtedly the knowledge on dispersal routes of *F. magna* on both continents. While the west-east and the south-north NA lineages corresponded with historical movement and current distribution of white-tailed deer, wapiti and caribou, the dispersal routes of *F. magna* in Europe agreed with the chronology of parasite findings based on the literature. Despite the occurrence of a few technical difficulties in the interpretation of microsatellite data (e.g. null alleles in some populations), microsatellites contributed to revealing population genetic patterns of *F. magna* that could not be achieved using mtDNA based methods. The employment of analytical methods based on different statistical presumptions (classical frequentist, Bayesian and coalescent-based) allowed relatively robust interpretation of the data, which generally agreed with the expectations based on host phylogeography and historical records on host movements [38, 39].

The giant liver fluke is a parasite with “never-ending story”; some facts of its ancestral history were known, while some remained unrevealed until recently. The very recent findings of the parasite in novel territories (e.g. Poland [55, 56] and Germany [57]) indicate that the distribution of *F. magna* in Europe is a dynamic and ongoing process. Therefore, future monitoring of the parasite distribution is necessary as a basis to prevent transmission to farmed and domestic ruminants. In addition to the veterinary importance, constant tracing of fascioloidosis will help us to maintain up-to-date status of the parasite occurrence.

## Additional files

Additional file 1: Table S1. Primer pairs and multiplex panels for 11 microsatellite loci applied in *Fascioloides magna* genotyping (modified according to Minárik et al. 2014). (DOCX 19 kb)
Additional file 2: Tables S4-S5. and **Figure S2.** Models of historical gene flow of *Fascioloides magna* populations tested in MIGRATE and estimates of relative population sizes. (DOCX 218 kb)
Additional file 3: Table S2. Summary of statistical parameters for 11 microsatellite loci of *Fascioloides magna* from North America. (DOCX 31 kb)
Additional file 4: Table S3. Summary of statistical parameters for 11 microsatellite loci of *Fascioloides magna* from Europe. (DOCX 26 kb)
Additional file 5: Figure S1. Implementation of delta K statistic and determination of number of genetic clusters using STRUCTURE HARVESTER. (DOCX 35 kb)

## Abbreviations

CR: Croatia
CZ: Czech Republic
CZ-PL: Czech Republic and south-western Poland
DFF: Danube floodplain forests
EU: Europe/European
GLR: Great Lakes region
He: Expected heterozygosity
Ho: Observed heterozygosity
HU: Hungary
HWE: Hardy-Weinberg equilibrium
IT: North-western Italy
LD: Linkage disequilibrium
mt: Mitochondrial
mtDNA: Mitochondrial DNA
NA: North America/North American
Na: Number of different alleles
Ne: Number of effective alleles
NPC: Northern Pacific coast
NQL: Northern Quebec and Labrador
PCoA: Principal coordinates analysis
PCR: Polymerase chain reaction
PL: Poland
RMT: Rocky Mountain trench
SAS: Gulf coast, lower Mississippi, and southern Atlantic seaboard
SK: Slovakia
SMM: Stepwise mutation model
STR: Short tandem repeats
TPM: Two-phase model
USA: Unites States of America
