# Supplementary material for: Population structure and dispersal routes of an invasive parasite, Fascioloides magna, in North America and Europe
Source: Parasit Vectors. 2016 Oct 13;9:547. doi: 10.1186/s13071-016-1811-z (PMC5064932; doi:10.1186/s13071-016-1811-z)
Supplement: Additional file 1: Table S1. — Primer pairs and multiplex panels for 11 microsatellite loci applied in Fascioloides magna genotyping (modified according to Minárik et al. 2014). (DOCX 19 kb) [file 13071_2016_1811_MOESM1_ESM.docx]

**Additional file 1. Table S1** Primer pairs and multiplex panels for 11 microsatellite loci applied in *Fascioloides magna* genotyping (modified

according to Minárik et al. 2014)

| **Locus** | **Forward primer (5̓ - 3̓)** | **Reverse primer (5̓ - 3̓)** | **Repeat**  **motif** | **GenBank**  **Acc. No.** | **Dye^a^** |
| --- | --- | --- | --- | --- | --- |
| Magna-F32 | F: CACTCGCCATTAACCAACTG | R: CAGACCAACAAAGAGGGTGAA | (tc)_n_ | KF856924 | YAKYE^1^ |
| Magna-F39 | F: TGACGAGAAAGGACAGTGGT | R: CATGAGCAGGTGTCATTCAGA | (tga)_n_ | KF856925 | AT550^2^ |
| Magna-F40 | F: AGGCGATGGACAGATAGAGG | R: CTCTCCCTCGTCCTCTCAGG | (ga)_n_ | KF856926 | AT565^1^ |
| Magna-F54 | F: CGTCGTGCCTGTATTGTTCC | R: GGTAACAAGAGGTTTCGGCA | (ac)_n_ | KF856927 | FAM^3^ |
| Magna-F81 | F: GCGAGCAAACAGTAAAGCA | R: GCTGCAATGTACTTTGTGCATGT | (ac)_n_ | KF856930 | FAM^3^ |
| Magna-F86 | F: ACTTAGGGGTTACGGTGCAG | R: AAGAACAAATACCACAGAGTATGACA | (ac)_n_ | KF856931 | AT550^2^ |
| Magna-F87 | F: ACTGCTTATTCTTTCATTCGTTGA | R: GATATACGGGATGAATGGCAA | (ac)_n_ | KF856932 | AT565^3^ |
| Magna-F90 | F: CACTCTCACCTGCCCTCTTC | R: TCCCATTTTCTTTGGGACAG | (ct)_n_ | KF856933 | FAM^4^ |
| Magna-F99 | F: TGTTCGACCCTCTGTTTTCC | R: ATTGCTGACCGACAAACCAT | (cg)_n_ | KF856934 | YAKYE^4^ |
| Magna-F101 | F: TGTGGTCTCGAAATTTGCAC | R: CGTGGAGGTAGACAAACGGT | (ca)_n_ | KF856935 | AT550^2^ |
| Magna-F107 | F: AAGAAGATGCGGTGGACTAAA | R: GACTGTTTGCACCGAACACA | (ag)_n_ | KF856936 | AT565^4^ |

^a^the multiplex PCR panel in which the primer pair was used
