# Supplementary material for: Population structure and dispersal routes of an invasive parasite, Fascioloides magna, in North America and Europe
Source: Parasit Vectors. 2016 Oct 13;9:547. doi: 10.1186/s13071-016-1811-z (PMC5064932; doi:10.1186/s13071-016-1811-z)

**Additional file 2** Models of historical gene flow of *Fascioloides magna*

populations tested in Migrate and estimates of relative population sizes

**Table S4** Migration patterns for North America and Europe

| ***Migration between all pairs***  **North America** | | | | | | | |  |  |  |  |
| --- | --- | --- | --- | --- | --- | --- | --- | --- | --- | --- | --- |
| **0** | Panmictic | |  |  |  |  |  |  |  |  |  |
| 1 |  |  | NQL  ↑ | 4 |  |  | NQL  ↑ | 7 |  |  | NQL  ↑ |
|  | NPC→RMT→GLR | | |  | NPC → RMT → GLR | | |  | NPC ← RMT ↔ GLR | | |
|  |  |  | ↑  SAS |  |  |  | ↓  SAS |  |  |  | ↑  SAS |
| 2 |  | NQL  ↑ | | **5** |  |  | **NQL**  **↑** | 8 | → → NQL ← ←  ↑ ↑ | | |
|  | NPC → RMT → GLR | | |  | **NPC → RMT ↔ GLR** | | |  | NPC RMT ↔ GLR | | |
|  |  |  | ↑  SAS |  |  |  | **↑**  **SAS** |  |  |  | ↑  SAS |
| 3 |  | → → NQL  ↑ ↑ | | 6 |  |  | NQL  ↑ | 9 | → → NQL  ↑ ↑ | | |
|  | NPC → RMT→ GLR | | |  | NPC ← RMT ← GLR | | |  | NPC RMT ↔ GLR | | |
|  |  |  | ↑  SAS |  |  |  | ↑  SAS |  |  |  | ↑  SAS |
| **Europe** | | |  |  |  |  |  |  |  |  |  |
| 0 | Panmictic | | | | | | |  |  |  |  |
| 1 | **CZ** → **SK** → **HU** → **CR** | | | | | | |  |  |  |  |
| 2 | CZ ← SK → HU → CR | | | | | | |  |  |  |  |
| 3 | CZ ← SK ← HU ← CR | | | | | | |  |  |  |  |

Direction of gene flow is indicated by arrows. Winning schemes with the highest posterior

probability are in bold.

**Table S5** Bayes factor (BF) ranking of hypotheses based on Bezier approximation scores

|  | **Raw**  **thermodynamic**  **score** | **Bezier**  **approximation**  **score** | **BF**  **probability** | **Model**  **ranking** |
| --- | --- | --- | --- | --- |
| **North America** | | | | |
| **5** | **-13436.94** | **-8712.74** | **1** | **1** |
| 2 | -13750.89 | -8856.59 | 1.13E-125 | 2 |
| 1 | -13927.64 | -8871.8 | 6.95E-139 | 3 |
| 7 | -13973.26 | -8907.79 | 3.82E-170 | 4 |
| 4 | -15413.21 | -8929.25 | 8.75E-189 | 5 |
| 3 | -14829.67 | -9096.23 | 0 | 6 |
| 6 | -15522.39 | -9131.89 | 0 | 7 |
| 8 | -16857.24 | -9137.35 | 0 | 8 |
| 9 | -38711.4 | -12827.11 | 0 | 9 |
| 0 | -97759.66 | -22580.33 | 0 | 10 |
| **Europe** | | | | |
| **1** | **-17887.07** | **-10493.75** | **1** | **1** |
| 3 | -20277.42 | -10690.02 | 3.33E-171 | 2 |
| 2 | -24910.92 | -11583.97 | 0 | 3 |
| 0 | -51844.3 | -16144.06 | 0 | 4 |

Winning schemes with the highest posterior probability are in bold.**Figure S2** Skyline plots for historical changes in Theta (*Θ*) obtained in Migrate

NPC


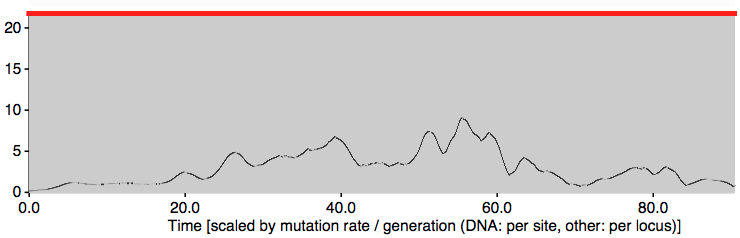


IT


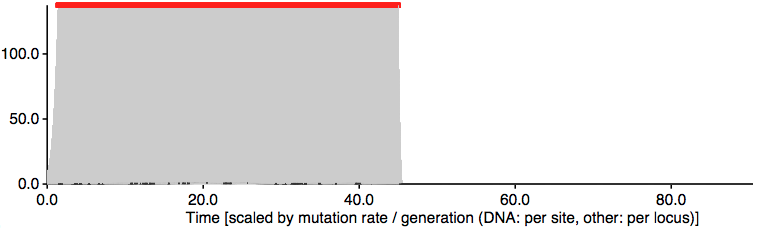


SC


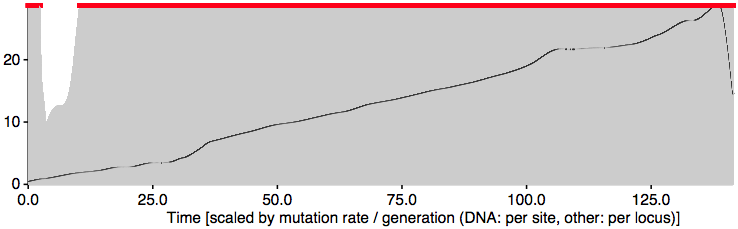


CZ


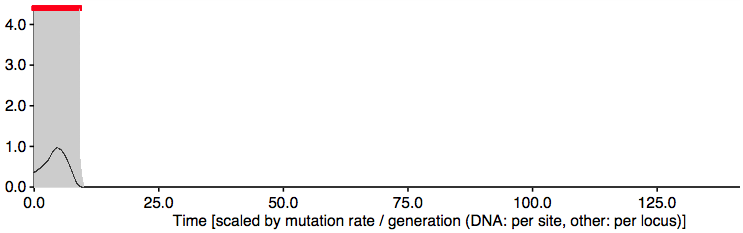


SK


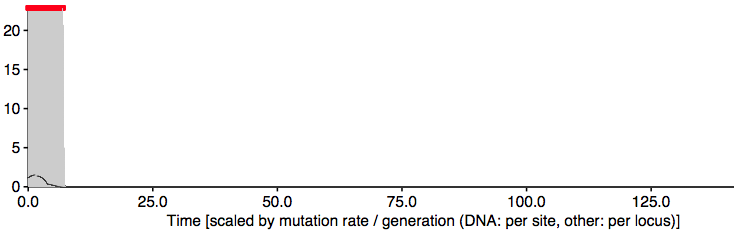


HU


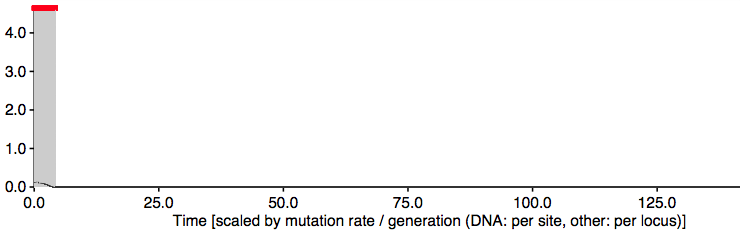


CR


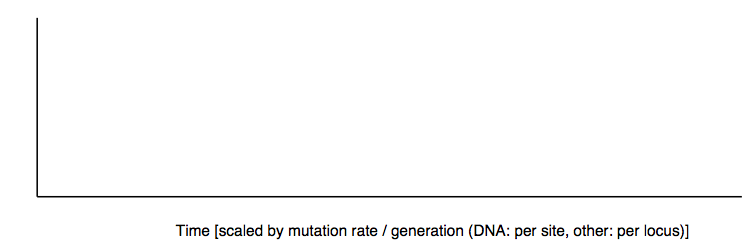

Supplement: Additional file 2: Tables S4-S5. — and Figure S2. Models of historical gene flow of Fascioloides magna populations tested in MIGRATE and estimates of relative population sizes. (DOCX 218 kb) [file 13071_2016_1811_MOESM2_ESM.docx]
