# Supplementary material for: Population structure and dispersal routes of an invasive parasite, Fascioloides magna, in North America and Europe
Source: Parasit Vectors. 2016 Oct 13;9:547. doi: 10.1186/s13071-016-1811-z (PMC5064932; doi:10.1186/s13071-016-1811-z)
Supplement: Additional file 3: Table S2. — Summary of statistical parameters for 11 microsatellite loci of Fascioloides magna from North America. (DOCX 31 kb) [file 13071_2016_1811_MOESM3_ESM.docx]

**Additional file 3. Table S2** Summary of statistical parameters for 11 microsatellite loci of *Fascioloides magna* from North America

| **Enzootic region** | **Locus** | **N** | **Na** | **Ne** | **Ho** | **uHe** | **F** | **DF** | **Signif** | **Null allele freq** |
| --- | --- | --- | --- | --- | --- | --- | --- | --- | --- | --- |
| **northern Pacific coast (NPC)** | Magna-F32 | 7 | 1 | 1.000 | 0 | 0 | n.a. | mono |  | 0 |
|  | Magna-F39 | 6 | 2 | 1.800 | 0 | 0.485 | 1.000 | 1 | * | 0.501 |
|  | Magna-F40 | 7 | 4 | 2.579 | 0.286 | 0.659 | 0.533 | 6 | ns | 0.203 |
|  | Magna-F54 | 7 | 1 | 1.000 | 0 | 0 | n.a. | mono |  | 0 |
|  | Magna-F81 | 7 | 2 | 1.508 | 0.429 | 0.363 | -0.273 | 1 | ns | 0 |
|  | Magna-F86 | 7 | 7 | 5.444 | 0.714 | 0.879 | 0.125 | 21 | ns | 0.056 |
|  | Magna-F87 | 7 | 3 | 2.333 | 0.857 | 0.615 | -0.500 | 3 | ns | 0 |
|  | Magna-F90 | 7 | 1 | 1.000 | 0 | 0 | n.a. | mono |  | 0 |
|  | Magna-F99 | 7 | 2 | 1.508 | 0.143 | 0.363 | 0.576 | 1 | ns | 0.145 |
|  | Magna-F101 | 7 | 3 | 1.815 | 0.286 | 0.484 | 0.364 | 3 | ns | 0.113 |
|  | Magna-F107 | 7 | 3 | 1.342 | 0.143 | 0.275 | 0.440 | 3 | ** | 0.089 |
| **Rocky Mountain trench (RMT)** | Magna-F32 | 89 | 2 | 1.046 | 0.045 | 0.044 | -0.023 | 1 | ns | 0 |
|  | Magna-F39 | 75 | 6 | 3.829 | 0.293 | 0.744 | 0.603 | 15 | *** | 0.472 |
|  | Magna-F40 | 88 | 13 | 3.171 | 0.500 | 0.689 | 0.270 | 78 | *** | 0.157 |
|  | Magna-F54 | 65 | 4 | 2.585 | 0.538 | 0.618 | 0.122 | 6 | *** | 0.467 |
|  | Magna-F81 | 89 | 4 | 1.928 | 0.382 | 0.484 | 0.206 | 6 | *** | 0.024 |
|  | Magna-F86 | 88 | 11 | 5.252 | 0.602 | 0.814 | 0.256 | 55 | *** | 0.119 |
|  | Magna-F87 | 89 | 9 | 2.480 | 0.517 | 0.600 | 0.134 | 36 | *** | 0.022 |
|  | Magna-F90 | 82 | 3 | 1.704 | 0.305 | 0.416 | 0.262 | 3 | *** | 0.235 |
|  | Magna-F99 | 89 | 4 | 2.834 | 0.573 | 0.651 | 0.115 | 6 | ** | 0 |
|  | Magna-F101 | 89 | 8 | 3.339 | 0.551 | 0.704 | 0.214 | 28 | *** | 0.072 |
|  | Magna-F107 | 88 | 4 | 2.879 | 0.602 | 0.656 | 0.077 | 6 | *** | 0.065 |
| **northern Quebec and Labrador (NQL)** | Magna-F32 | 13 | 2 | 1.166 | 0.154 | 0.148 | -0.083 | 1 | ns | 0 |
|  | Magna-F39 | 13 | 1 | 1.000 | 0 | 0 | n.a. | mono |  | 0 |
|  | Magna-F40 | 13 | 5 | 2.331 | 0.462 | 0.594 | 0.192 | 10 | ns | 0.070 |
|  | Magna-F54 | 11 | 1 | 1.000 | 0 | 0 | n.a. | mono |  | 0.392 |
|  | Magna-F81 | 13 | 2 | 1.166 | 0.154 | 0.148 | -0.083 | 1 | ns | 0 |
|  | Magna-F86 | 13 | 6 | 2.432 | 0.846 | 0.612 | -0.437 | 15 | ns | 0 |
|  | Magna-F87 | 13 | 4 | 3.347 | 0.846 | 0.729 | -0.207 | 6 | ns | 0 |
|  | Magna-F90 | 13 | 1 | 1.000 | 0 | 0 | n.a. | mono |  | 0 |
|  | Magna-F99 | 13 | 2 | 1.827 | 0.385 | 0.471 | 0.150 | 1 | ns | 0.047 |
|  | Magna-F101 | 13 | 7 | 4.568 | 0.615 | 0.812 | 0.212 | 21 | ns | 0.093 |
|  | Magna-F107 | 13 | 2 | 1.550 | 0.462 | 0.369 | -0.300 | 1 | ns | 0 |
| **Great Lakes region (GLR)** | Magna-F32 | 14 | 2 | 1.508 | 0.429 | 0.349 | -0.273 | 1 | ns | 0 |
|  | Magna-F39 | 13 | 5 | 2.504 | 0.231 | 0.625 | 0.616 | 10 | ** | 0.350 |
|  | Magna-F40 | 14 | 11 | 7.000 | 0.786 | 0.889 | 0.083 | 55 | ns | 0.039 |
|  | Magna-F54 | 13 | 3 | 2.024 | 0.385 | 0.526 | 0.240 | 3 | ns | 0.247 |
|  | Magna-F81 | 14 | 7 | 5.939 | 0.643 | 0.862 | 0.227 | 21 | ns | 0.103 |
|  | Magna-F86 | 14 | 7 | 5.444 | 0.786 | 0.847 | 0.038 | 21 | * | 0.017 |
|  | Magna-F87 | 14 | 7 | 5.444 | 0.857 | 0.847 | -0.050 | 21 | ns | 0 |
|  | Magna-F90 | 10 | 2 | 1.220 | 0 | 0.189 | 1.000 | 1 | ** | 0.574 |
|  | Magna-F99 | 14 | 3 | 2.405 | 0.643 | 0.606 | -0.100 | 3 | ns | 0 |
|  | Magna-F101 | 14 | 7 | 3.015 | 0.786 | 0.693 | -0.176 | 21 | ns | 0 |
|  | Magna-F107 | 14 | 6 | 4.000 | 0.929 | 0.778 | -0.238 | 15 | ns | 0 |
| **Gulf coast, lower Mississippi and southern Atlantic seaboard (SAS)** | Magna-F32 | 53 | 3 | 1.958 | 0.566 | 0.494 | -0.157 | 3 | * | 0 |
|  | Magna-F39 | 52 | 6 | 2.852 | 0.212 | 0.656 | 0.674 | 15 | *** | 0.299 |
|  | Magna-F40 | 50 | 11 | 5.974 | 0.860 | 0.841 | -0.033 | 55 | *** | 0.109 |
|  | Magna-F54 | 53 | 3 | 1.636 | 0.094 | 0.392 | 0.757 | 3 | *** | 0.212 |
|  | Magna-F81 | 52 | 5 | 1.807 | 0.385 | 0.451 | 0.139 | 10 | ns | 0.122 |
|  | Magna-F86 | 53 | 15 | 8.072 | 0.566 | 0.884 | 0.354 | 105 | *** | 0.165 |
|  | Magna-F87 | 53 | 11 | 5.584 | 0.642 | 0.829 | 0.219 | 55 | *** | 0.099 |
|  | Magna-F90 | 53 | 3 | 2.038 | 0.358 | 0.514 | 0.296 | 3 | ns | 0.100 |
|  | Magna-F99 | 53 | 2 | 1.931 | 0.245 | 0.487 | 0.491 | 1 | *** | 0.160 |
|  | Magna-F101 | 53 | 9 | 4.773 | 0.660 | 0.798 | 0.165 | 36 | *** | 0.073 |
|  | Magna-F107 | 53 | 7 | 4.448 | 0.623 | 0.783 | 0.197 | 21 | *** | 0.086 |

*N* sample size, *Na* number of different alleles, *Ne* number of effective alleles, *Ho* observed heterozygosity, *uHe* unbiased expected heterozygosity, *F* fixation index, *n.a*. not available, *DF* degrees of freedom, *mono* monomorphic locus, *Signif* significance values for Chi-Square Tests for Hardy-Weinberg Equilibrium (ns=not significant, * P<0.05, ** P<0.01, *** P<0.001), and *Null allele freq* frequency of null alleles estimated in MicroChecker using Brookfield (1996) method
