# Supplementary material for: Population structure and dispersal routes of an invasive parasite, Fascioloides magna, in North America and Europe
Source: Parasit Vectors. 2016 Oct 13;9:547. doi: 10.1186/s13071-016-1811-z (PMC5064932; doi:10.1186/s13071-016-1811-z)
Supplement: Additional file 4: Table S3. — Summary of statistical parameters for 11 microsatellite loci of Fascioloides magna from Europe. (DOCX 26 kb) [file 13071_2016_1811_MOESM4_ESM.docx]

**Additional file 4. Table S3** Summary of statistical parameters for 11 microsatellite loci of *Fascioloides magna* from Europe

| **Natural foci** | **Locus** | **N** | **Na** | **Ne** | **Ho** | **uHe** | **F** | **DF** | **Signif** | **Null allele freq** |
| --- | --- | --- | --- | --- | --- | --- | --- | --- | --- | --- |
| **Italy (IT)** | Magna-F32 | 51 | 4 | 1.061 | 0.039 | 0.058 | 0.320 | 6 | *** | 0.017 |
|  | Magna-F39 | 47 | 1 | 1.000 | 0 | 0 | n.a. | mono |  | 0.280 |
|  | Magna-F40 | 38 | 4 | 3.526 | 0.737 | 0.726 | -0.029 | 6 | ** | 0.383 |
|  | Magna-F54 | 42 | 3 | 1.768 | 0.238 | 0.440 | 0.452 | 3 | * | 0.430 |
|  | Magna-F81 | 51 | 2 | 1.756 | 0.431 | 0.435 | -0.002 | 1 | ns | 0 |
|  | Magna-F86 | 51 | 9 | 3.551 | 0.784 | 0.725 | -0.092 | 36 | ns | 0 |
|  | Magna-F87 | 51 | 3 | 2.751 | 0.686 | 0.643 | -0.078 | 3 | ns | 0 |
|  | Magna-F90 | 51 | 1 | 1.000 | 0 | 0 | n.a. | mono |  | 0 |
|  | Magna-F99 | 51 | 2 | 1.310 | 0.275 | 0.239 | -0.159 | 1 | ns | 0 |
|  | Magna-F101 | 51 | 3 | 1.354 | 0.294 | 0.264 | -0.124 | 3 | ns | 0 |
|  | Magna-F107 | 51 | 2 | 1.215 | 0.196 | 0.179 | -0.109 | 1 | ns | 0 |
| **Czech Republic and south- western Poland**  **(CZ-PL)** | Magna-F32 | 105 | 6 | 3.049 | 0.838 | 0.675 | -0.247 | 15 | *** | 0 |
|  | Magna-F39 | 105 | 10 | 4.649 | 0.838 | 0.789 | -0.068 | 45 | *** | 0.134 |
|  | Magna-F40 | 103 | 8 | 5.411 | 0.660 | 0.819 | 0.190 | 28 | *** | 0.129 |
|  | Magna-F54 | 103 | 3 | 1.147 | 0.068 | 0.129 | 0.470 | 3 | *** | 0.155 |
|  | Magna-F81 | 104 | 3 | 2.676 | 0.433 | 0.629 | 0.309 | 3 | *** | 0.146 |
|  | Magna-F86 | 105 | 7 | 4.218 | 0.790 | 0.767 | -0.036 | 21 | ns | 0 |
|  | Magna-F87 | 105 | 9 | 6.314 | 0.743 | 0.846 | 0.117 | 36 | *** | 0.054 |
|  | Magna-F90 | 90 | 3 | 2.656 | 0.144 | 0.627 | 0.768 | 3 | *** | 0.482 |
|  | Magna-F99 | 105 | 3 | 2.440 | 0.638 | 0.593 | -0.081 | 3 | ns | 0 |
|  | Magna-F101 | 102 | 5 | 2.435 | 0.539 | 0.592 | 0.085 | 10 | ns | 0.126 |
|  | Magna-F107 | 105 | 6 | 3.769 | 0.695 | 0.738 | 0.054 | 15 | *** | 0.023 |
| **Danube floodplain forests (DFF)** | Magna-F32 | 100 | 5 | 1.595 | 0.440 | 0.375 | -0.179 | 10 | *** | 0 |
|  | Magna-F39 | 100 | 3 | 1.118 | 0.050 | 0.106 | 0.526 | 3 | *** | 0.050 |
|  | Magna-F40 | 97 | 5 | 2.312 | 0.557 | 0.570 | 0.019 | 10 | * | 0.115 |
|  | Magna-F54 | 100 | 2 | 1.220 | 0.020 | 0.181 | 0.889 | 1 | *** | 0.136 |
|  | Magna-F81 | 100 | 3 | 2.418 | 0.550 | 0.589 | 0.062 | 3 | ns | 0.023 |
|  | Magna-F86 | 100 | 3 | 2.160 | 0.550 | 0.540 | -0.024 | 3 | ns | 0 |
|  | Magna-F87 | 100 | 7 | 3.954 | 0.610 | 0.751 | 0.184 | 21 | * | 0.079 |
|  | Magna-F90 | 99 | 3 | 2.005 | 0.273 | 0.504 | 0.456 | 3 | *** | 0.181 |
|  | Magna-F99 | 100 | 3 | 2.600 | 0.580 | 0.619 | 0.058 | 3 | ns | 0.022 |
|  | Magna-F101 | 100 | 3 | 1.815 | 0.470 | 0.451 | -0.047 | 3 | ns | 0 |
|  | Magna-F107 | 100 | 3 | 1.573 | 0.310 | 0.366 | 0.149 | 3 | ns | 0.040 |

*N* sample size, *Na* number of different alleles, *Ne* number of effective alleles, *Ho* observed heterozygosity, *uHe* unbiased expected heterozygosity, *F* fixation index, *n.a.* not available, *DF* degrees of freedom, *mono* monomorphic locus, *Signif* significance values for Chi-Square Tests for Hardy-Weinberg Equilibrium (ns=not significant, * P<0.05, ** P<0.01, *** P<0.001), and *Null allele freq* frequency of null alleles estimated in MicroChecker using Brookfield (1996) method
