# Supplementary material for: Population structure and dispersal routes of an invasive parasite, Fascioloides magna, in North America and Europe
Source: Parasit Vectors. 2016 Oct 13;9:547. doi: 10.1186/s13071-016-1811-z (PMC5064932; doi:10.1186/s13071-016-1811-z)
Supplement: Additional file 5: Figure S1. — Implementation of delta K statistic and determination of number of genetic clusters using STRUCTURE HARVESTER. (DOCX 35 kb) [file 13071_2016_1811_MOESM5_ESM.docx]

**Additional file 5. Figure S1** Implementation of delta K statistic and determination of number of genetic clusters using Structure Harvester

ΔK

ΔK

K

| **2752.946** | 0.732 | **5.102** | 2.192 | 0.108 | 2.054 | **70.419** | 0.199 |
| --- | --- | --- | --- | --- | --- | --- | --- |
